# Risk factors for lower limb lymphedema in gynecologic cancer patients after initial treatment

**DOI:** 10.1007/s10147-019-01608-6

**Published:** 2020-01-06

**Authors:** Teruyo Kunitake, Tatsuyuki Kakuma, Kimio Ushijima

**Affiliations:** 1 Clinical Research Center, Kurume University Hospital, 67 Asahi-machi, Kurume-shi, Fukuoka 830-0011 Japan; 2 Kurume University Graduate School of Medicine Kurume University Graduate School of Medicine Graduate School Biostatistics, Fukuoka, Japan; 3 Department of Biostatistics Center, Medical School, Kurume University, Fukuoka, Japan; 4 Department of Obstetrics and Gynecology, Kurume University School of Medicine, Kurume, Fukuoka Japan

**Keywords:** Lower limb lymphedema, Risk factor, Gynecologic cancer

## Abstract

**Background:**

Most studies on lower limb lymphedema have been conducted in gynecologic cancer patients who underwent surgery for gynecologic malignancy. This study aimed to evaluate the risk factors for lower limb lymphedema development in gynecologic cancer patients who underwent initial treatment.

**Methods:**

A retrospective cohort design was used to follow 903 gynecologic cancer patients who underwent treatment at Kurume University Hospital between January 1, 2013 and December 31, 2015. Data analyses were performed in 356 patients, and the patients were followed up until December 31, 2017. The model comprised two components to facilitate statistical model construction. Specifically, a discrete survival time model was constructed, and a complementary log–log link model was fitted to estimate the hazard ratio. Associations between risk factors were estimated using generalized structural models.

**Results:**

The median follow-up period was 1083 (range 3–1819) days, and 54 patients (15.2%) developed lower limb lymphedema, with a median onset period of 240 (range 3–1415) days. Furthermore, 38.9% of these 54 patients developed lower limb lymphedema within 6 months and 85.2% within 2 years. International Federation of Gynecology and Obstetrics stage, radiotherapy, and number of lymph node dissections (≥ 28) were significant risk factors.

**Conclusion:**

Simultaneous examination of the relationship between lower limb lymphedema and risk factors, and analysis among the risk factors using generalized structural models, enabled us to construct a clinical model of lower limb lymphedema for use in clinical settings to alleviate this condition and improve quality of life.

## Introduction

Most research on lower limb lymphedema (LLL) has been conducted in gynecologic cancer patients after surgery for gynecologic malignancy. Although studies on the causes of LLL in Japan and abroad have been conducted in recent years, research is still lagging compared to that on upper limb lymphedema [1, 2]. Patients with LLL suffer psychological damage due to motor function disorders and physical handicapping and may develop severe complications, such as cellulitis, which significantly worsen quality of life (QOL).

In previous studies, postoperative radiotherapy [3–13] and the number of lymph node (LN) dissections [9, 10, 12–19] were recognized as risk factors for LLL; however, reported risk factors differ between studies [20, 21]. Although lymphedema development appeared in a literature review, statistical analysis should simultaneously include the relationship between lymphedema onset and risk factors, and the relationship between risk factors, to determine the risk factors for lymphedema. To date, no study has included a prediction model including the relationship between risk factors [1, 22].

This study aimed to estimate the “survival curve” of LLL in gynecologic cancer patients after initial treatment using 5-year follow-up records, and to construct a clinical pathology model of LLL development based on structural equation modeling where risk factors associated with LLL incidence and clinically interpretable relationships between risk factors can be simultaneously examined.

## Patients and methods

### Study design and data source

**Fig. 1:**
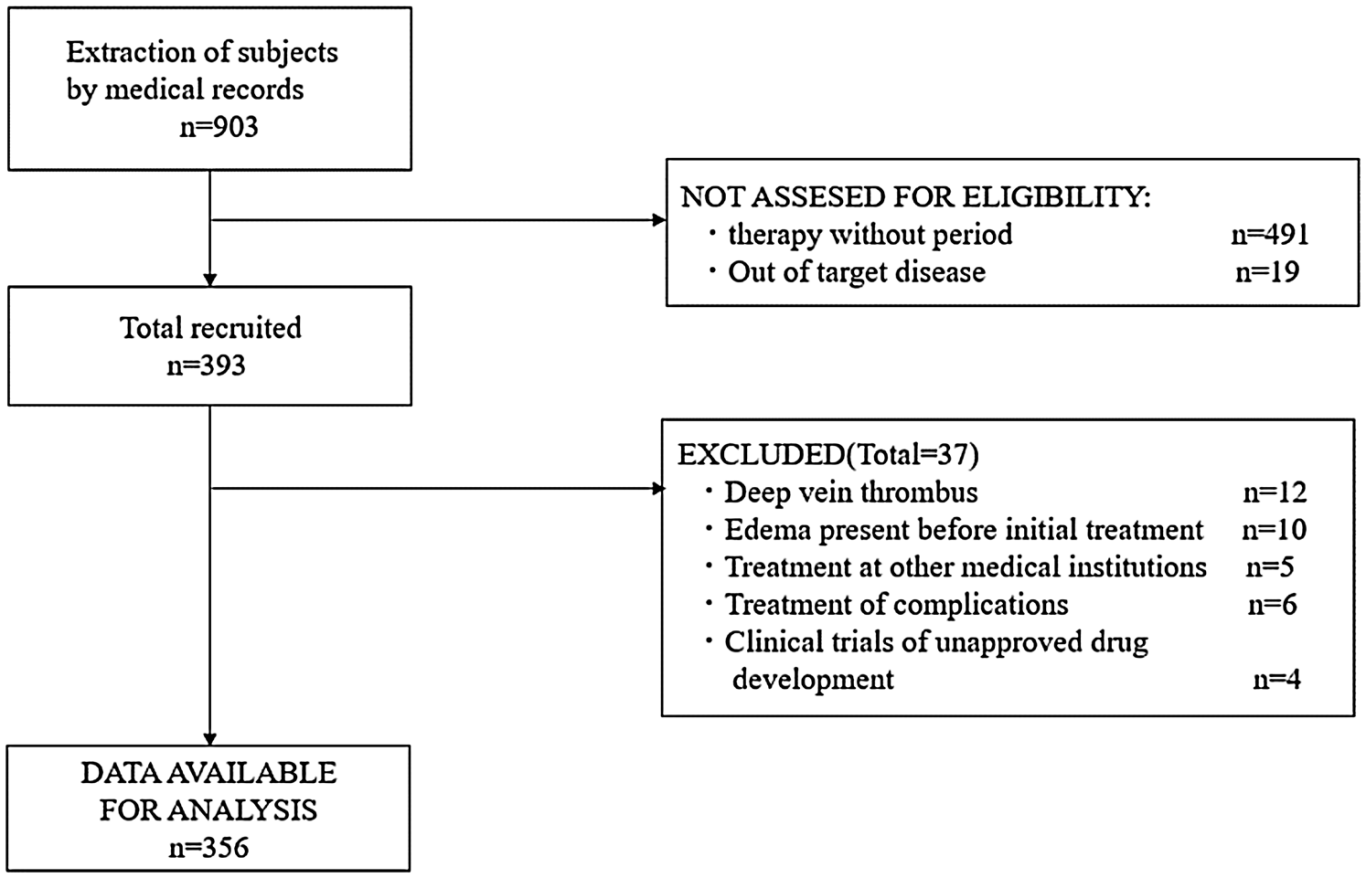
Subject selection and follow-up flow diagram

A retrospective cohort design was used to follow-up 903 gynecologic cancer (cervical, endometrial, ovarian, and fallopian tube) patients who underwent treatment at the Kurume University Hospital Gynecology Department between January 1, 2013 and December 31, 2015. Finally, after a 3-year accrual period, data of 356 patients were used for analyses, and patients were followed up until December 31, 2017. Patients who underwent initial treatment for gynecologic cancer, including surgery, chemotherapy, radiotherapy, or any combination, were included. The exclusion criteria are described in Fig. 1.

The presence or absence and time of onset of LLL depended on the judgment of the attending physician. The physician in charge diagnosed LLL through physical examinations, including those for the presence of left-right difference in lower limb thickness. This study was conducted with the approval of the Kurume University Ethics Committee (number: 14063).

### Risk factors

Data on 19 risk factors were obtained from hospital medical records; they were grouped into internal and external risk factors to facilitate the construction of statistical models, as shown in Table 1. Patients’ age and body mass index (BMI) were recorded at initial treatment.

**Table 1:** Possible risk factors used for statistical analysis

Risk factors group	Risk factors
Internal risk factors	
Patient background	Age^a^, BMI^a^, complication and medical history^b^, family history^b^, delivery history^a^
Disease progress status	Type of gynecological cancer^c^, FIGO staging^c^, lymph node metastasis
External risk factors	
Method of treatment	Surgery, chemotherapy, radiotherapy, type of therapy
Operative therapy relation	Type of surgery, number of LN resection, inguinal LN dissection, pelvic LN biopsy, pelvic LN dissection, para-aortic LN biopsy, para-aortic LN dissection

*BMI* body mass index, *FIGO* International Federation of Gynecology and Obstetrics, *LN* lymph node, *CI* confidence interval

^a^At the time of initial treatment, if height and weight data do not exist, data at the time closest to the initial treatment day are adopted

^b^Data on the occurrence of diabetes, hypertension, and other cancers

^c^If the diagnosis results differ before and after treatment, the diagnosis after treatment regarding endometrial and ovarian cancer

### Statistical analysis

Risk factors were dichotomized to facilitate the interpretation of model parameters. Age was dichotomized with patients’ mean age value at initial treatment. BMI, an index of obesity, was dichotomized as ≥ 25 kg/m^2^, using a clinically reasonable cut-off value. Cases were categorized according to the International Federation of Gynecology and Obstetrics (FIGO) guidelines into two categories by severity: stages I–II and stages III–IV. A tree model was applied as a method of binary conversion of LN clearance and the number of LNs dissected was categorized as ≥ 28 and < 28. In a previous study [8], 31 LN dissections were used as data cut-off values, and in this study, 28 or more were determined to be appropriate cutoff values. In clinical practice, LN biopsy or LN dissection is selected depending on the risk of cancer LN metastasis. Thus, the number of LN varies, depending on the location, but the number of LN dissections cannot be adjusted in each area. Therefore, the value for each LN area was set to two values that performed or not performed.

For univariate analysis, the Cox proportional hazard model was used to estimate the hazard ratio of each risk factor on the incidence rate of LLL. Multiple complex relationships between risk factors affect the incidence of LLL [1]. To model the complexity of LLL development, the structural equation modeling approach was employed to examine the impact of the risk factors. The model comprised components; the first one is the survival model which estimated the hazard of risk factors, and the second one represented the complex relationship between risk factors. Specifically, the discrete survival time model was constructed by partitioning the follow-up period into 180-day intervals. Additionally, the complementary log–log link model [23] was fitted to estimate hazard ratio, while the association between risk factors was estimated using generalized structural models (GSEMs). The outline of the GSEM is described in the appendix. Data analysis was performed using the SAS 9.4 TS Level 1 M3 statistical software (SAS Institute Inc., NC, USA), JMP Pro13.2.1 (JMP is a product of SAS, located at A Campus Drive, Cray, NC, USA), Stata/MP 14.0 (StataCorp LP 4905 Lakeway Drive College Station, TX 77845 USA), and the R version 2.15.2 (The R Foundation for Statistical Computing, Vienna, Austria).

## Results

Table 2 shows patient characteristics categorized by type of gynecologic cancer. All 356 patients were Japanese, with a mean age of 58.4 years (median 59.0 years; range 18–95 years) and mean BMI of 23.2 kg/m^2^ (median 22.4 kg/m^2^; range 14.1–47.6 kg/m^2^). Mean age at first treatment (median, range) for cervical carcinoma, endometrial carcinoma, and ovarian cancer, including fallopian tube cancer, was 54.8 years (median 49 years; range 30–81 years), 61.6 years (median 59.5 years; range 44–86 years), and 57.7 years (median 60 years; range 46–83 years), respectively. FIGO for staging gynecological cancer was 63% during stage I (225 patients) and 17 % during stage III (61 patients). The types of therapy were surgery (34 patients), chemotherapy and radiotherapy (29 patients), radiotherapy (18 patients), or surgery and radiotherapy (17 patients). Specifically, there are two types of radiotherapy: external beam irradiation and intracavitary brachytherapy. Endometrial or ovarian cancer is externally irradiated, and cervical cancer is irradiated externally or with radiotherapy combined with external and intracavitary brachytherapy. External beam irradiation can irradiate the lesion, uterus, vagina, and pelvic LN area from outside the body. However, intracavitary brachytherapy can be performed by concentrating a high dose at the lesion site using a radiation source from the uterus or vagina, while suppressing the radiation dose of other organs. There are differences in the type of radiotherapy depending on the type of cancer, such as external beam irradiation for endometrial/ovarian cancer, and external beam irradiation after surgery or intracavitary brachytherapy combined with externa beam irradiation. Although detailed data are not shown, differences in radiotherapy by cancer type were not found to directly affect the development of LLL (OR 0.79; *p* = 0.56). External beam radiation was performed for the entire pelvis; total dose was 45–50.4 Gy (1.8 Gy, 25–28 Fr). Conversely, surgery and a combination of surgery and chemotherapy were performed on 90% of patients with endometrial and ovarian cancer.

**Table 2:** Patient characteristics by type of gynecological cancer

Characteristics	Cervical	Endometrial	Ovarian and tube	All
	*N* = 121 (34%)	*N* = 151 (42.4%)	*N* = 84 (23.6%)	*N* = 356 (100%)
Age^a^ (year), mean ± SD	54.8 ± 15	61.6 ± 10.7	57.7 ± 14.8	58.4 ± 13.6
BMI^a^ (kg/m^2^), mean ± SD	22.0 ± 4.0	24.7 ± 4.6	21.9 ± 4.3	23.2 ± 4.5
Stage (FIGO), *n* (%)				
I	73 (60%)	113 (75%)	39 (47%)	225 (63%)
II	25 (21%)	7 (4%)	11 (13%)	43 (12%)
III	13 (11%)	21 (14%)	27 (32%)	61 (17%)
IV	10 (8%)	10 (7%)	7 (8%)	27 (8%)
Type of therapy, *n* (%)^b^				
Sur only	34 (28%)	78 (52%)	27 (33%)	139 (39%)
Chemo only	2 (2%)	0 (0%)	1 (1%)	3 (1%)
Rad only	18 (15%)	0 (0%)	0 (0%)	18 (5%)
Sur and Chemo	14 (11%)	62 (41%)	54 (64%)	130 (36%)
Chemo and Rad	29 (24%)	1 (1%)	1 (1%)	31 (9%)
Sur and Rad	17 (14%)	5 (3%)	0 (0%)	22 (6%)
Sur and Chemo and Rad	7 (6%)	5 (3%)	1 (1%)	13 (4%)
Number of LN resections				
Mean ± SD	27.5 ± 15.7	22.8 ± 18.8	23.2 ± 20.5	24.0 ± 18.7
Min–Max	0–67	0–88	0–97	0–97

*Sur* surgery, *Chemo* chemotherapy, *Rad* radiotherapy, *SD* standard deviation, *BMI* body mass index, *FIGO* International Federation of Gynecology and Obstetrics, *LN* lymph node, *Min* minimum values, *Max* maximum values

^a^At the time of initial treatment

^b^The type of treatment is the treatment received between the initial treatment after the diagnosis of a gynecologic malignancy and the end of the follow-up after diagnosis of a gynecological malignancy and the end of follow-up

**Fig. 2:**
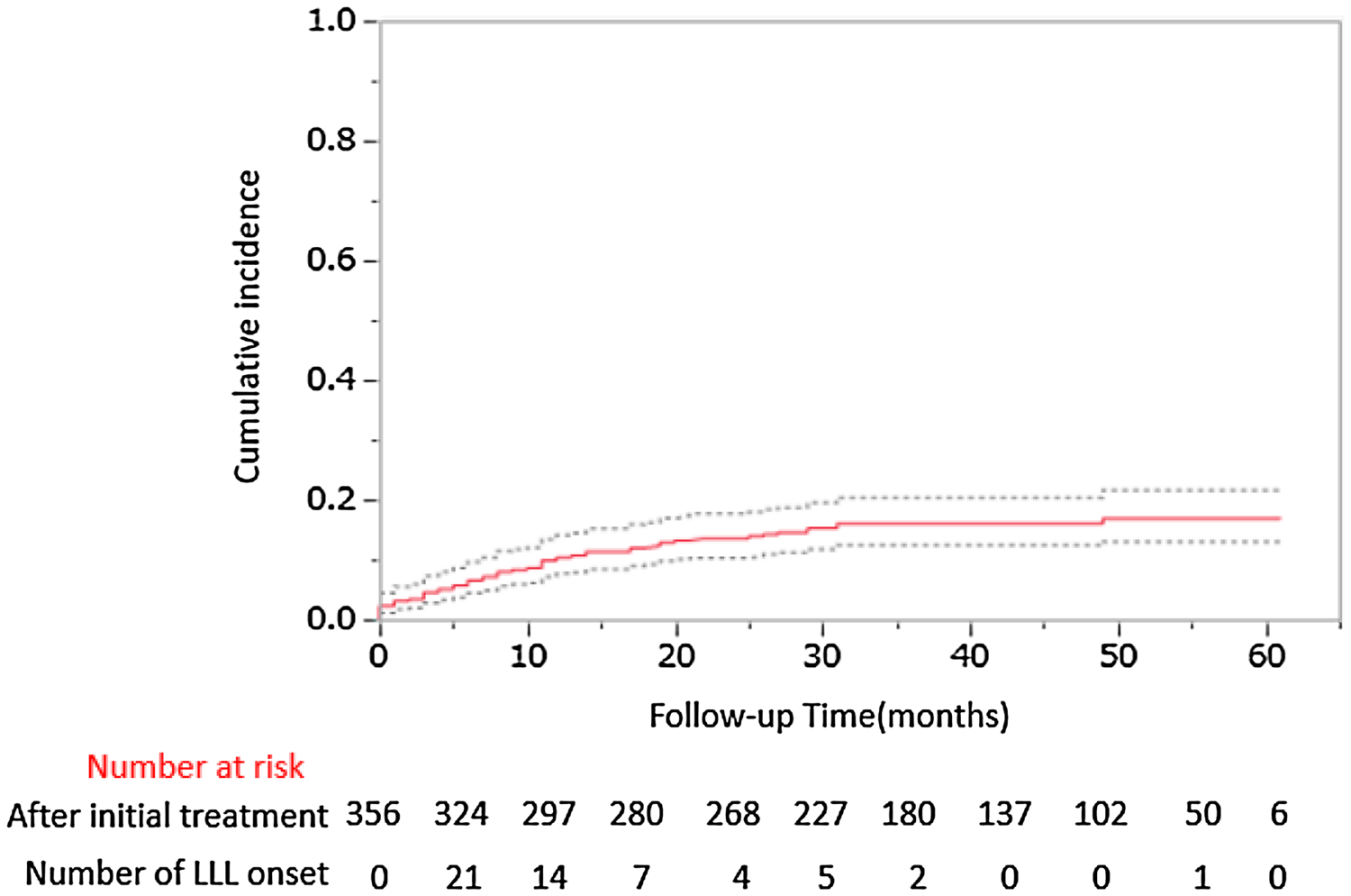
Kaplan–Meier of plot of the cumulative incidence of lower limb lymphedema (LLL)

The Kaplan–Meier survival curve is shown in Fig. 2 where the median follow-up period for 356 patients was 1083 days (range 3–1819 days). A total of 54 patients (15.2%) developed LLL with a median onset period of 240 days (range 3–1415 days). Furthermore, 38.9% of them developed LLL within 6 months and 85.2% within 2 years. In this study, the follow-up period was set from 720 to 1800 days, but LLL occurred 3 days after the first treatment. Also, seven out of 302 patients who did not develop LLL had a follow-up period of less than 100 days, for reasons such as transfer after initial treatment.

**Fig. 3:**
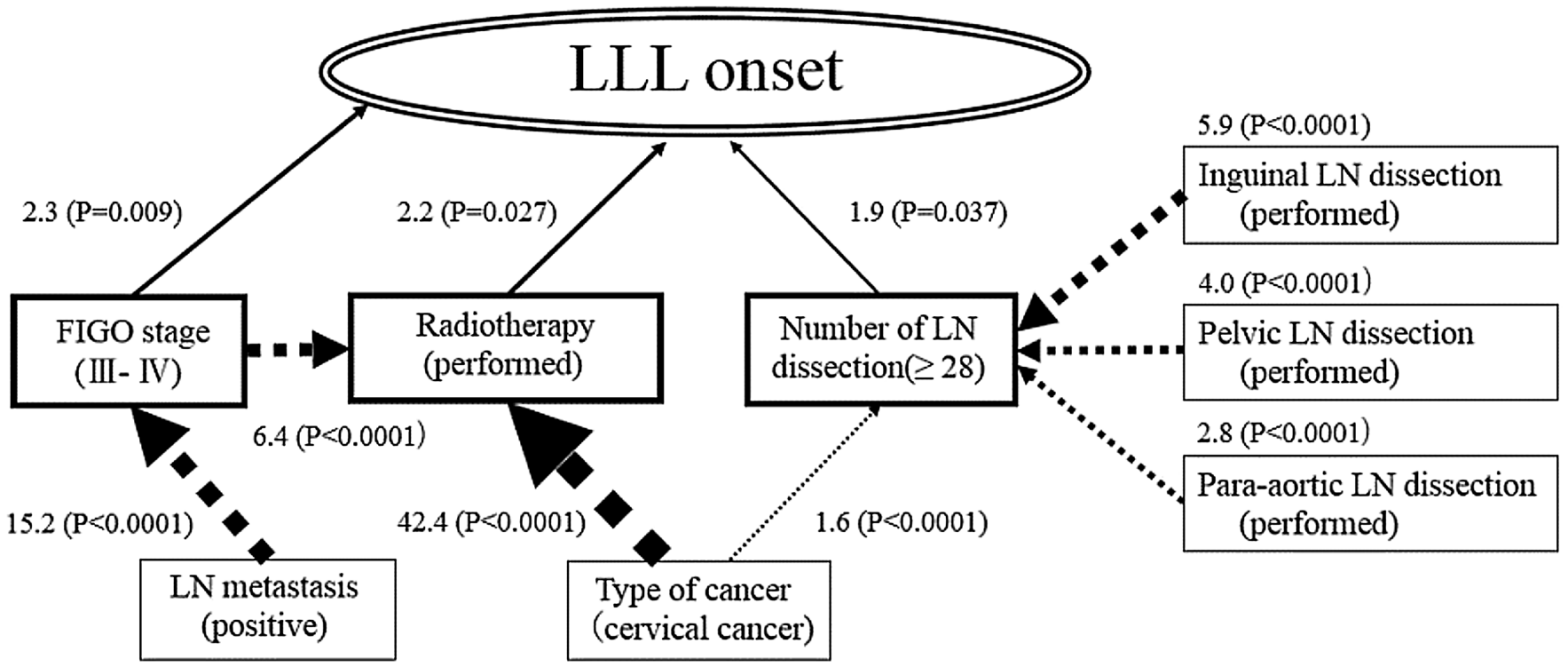
Risk factor model of lower-limb lymphedema (LLL). The solid arrow is the hazard ratio. The thickness of the solid arrow expresses the strength of the relationship. The dotted arrow is the odds ratio. *FIGO* International Federation of Gynecology and Obstetrics, *LN* lymph node

Table 3 shows the distribution of risk factors among patients with and without LLL. The hazard ratio for each risk factor was obtained from a univariate Cox proportional hazard model, and 95% confidence intervals are also shown in Table 3. FIGO stage (III–IV), radiotherapy, and number of LN dissections (≥ 28) were significant, with the level of significance being less than 5%. Generalized structure equation modeling was employed to construct a clinical model as shown in Fig. 3. This clinical model consisted of two components. The first was a discrete time survival model to estimate the hazard ratio among risk factors. FIGO stage (HR 2.3; 95% CI 1.2–4.2), radiotherapy (HR 2.2; 95% CI 1.1–4.5), and the number of LNs dissected (≥ 28) (HR 1.9; 95% CI 1.0–3.5) were found to be significant risk factors for LLL development. Cancer types, inguinal LN dissection, pelvic LN dissection, para-aortic LN dissection, and LN metastasis have an indirect effect on the onset of LLL and have no direct effects.

**Table 3:** Influence of factors on the absence or presence of lower limb lymphedema

Variables	Variable category	Absence	Presence	Hazard ratio 95% CI
		*N* = 302 (84.8%)	*N* = 54 (15.2%)	
Age (years), *n* (%)	≥ 58	168 (55.6)	30 (55.6)	0.59–1.73
	< 58 [reference]	134 (44.4)	24 (44.4)	
BMI (kg/m^2^), *n* (%)	≥ 25	86 (28.5)	13 (24.1)	0.44–1.54
	< 25 [reference]	216 (71.5)	41 (75.9)	
Medical history and complication^a^, *n* (%)	Yes	112 (37.5)	15 (27.8)	0.38–1.25
	No [reference]	187 (62.5)	39 (72.2)	
Family history, *n* (%)	Yes	56 (18.5)	9 (16.7)	0.44–1.84
	No [reference]	246 (81.5)	45 (83.3)	
Pregnancy^a^, *n* (%)	Yes	249 (83.3)	42 (77.8)	0.37–1.33
	No [reference]	50 (16.7)	12 (22.2)	
Stage (FIGO), *n* (%)	III–IV	66 (21.9)	22 (40.7)	1.42–4.20
	I–II [reference]	236 (78.2)	32 (59.3)	
Type of cancer, *n* (%)	Cervical	100 (33.1)	21 (38.9)	0.51–1.91
	Endometrial	133 (44.0)	18 (33.3)	0.33–1.31
	Ovarian and tube [reference]	69 (22.9)	15 (27.8)	
Surgery, *n* (%)	Yes	260 (86.1)	44 (81.5)	0.35–1.38
	No [reference]	42 (13.9)	10 (18.6)	
Chemotherapy, *n* (%)	Yes	153 (50.7)	24 (44.4)	0.45–1.32
	No [reference]	149 (49.3)	30 (55.6)	
Radiotherapy, *n* (%)	Yes	65 (22.0)	19 (35.2)	1.09–3.35
	No [reference]	237 (78.0)	35 (64.8)	
Type of therapy, *n* (%)	Combination therapy	166 (55.0)	31 (57.4)	0.57–1.67
	Monotherapy [reference]	136 (45.0)	23 (42.6)	
Type of surgery^b^, *n* (%)	Total hysterectomy	177 (76.3)	29 (76.3)	0.12–6.55
	Modified radical hysterectomy	8 (3.5)	2 (5.3)	0.11–13.14
	Radical hysterectomy	42 (18.1)	6 (15.8)	0.09–6.28
	Others [reference]	5 (2.2)	1 (2.6)	
Inguinal LN dissection, *n* (%)	Yes	137 (52.7)	29 (65.9)	0.87–3.04
	No [reference]	123 (47.3)	15 (34.1)	
Pelvic LN biopsy, *n* (%)	Yes	52 (20)	6 (13.6)	0.28–1.58
	No [reference]	208 (80)	38 (86.4)	
Pelvic LN dissection, *n* (%)	Yes	166 (63.8)	34 (77.3)	0.86–3.53
	No [reference]	94 (36.2)	10 (22.7)	
Para-aortic LN biopsy, *n* (%)	Yes	94 (36.2)	16 (36.4)	0.53–1.79
	No [reference]	166 (63.9)	28 (63.6)	
Para-aortic LN dissection, *n* (%)	Yes	68 (26.2)	15 (34.1)	0.75–2.60
	No [reference]	192 (73.9)	29 (65.9)	
Number of LN dissection^c^, *n* (%)	≥ 28	95 (36.8)	25 (36.8)	1.14–3.77
	< 28 [reference]	163 (63.2)	19 (43.2)	
LN metastasis	Positive	41 (15.8)	11 (25.0)	0.89–3.49
	Negative [reference]	219 (84.2)	33 (75.0)	

Total hysterectomy is the routine procedure for endometrial cancer, ovarian cancer, severe dysplasia, or invasive cervical cancer stage IA1. Bilateral oophorectomy is usually performed. Also, pelvic or para-aortic lymph node dissection is also performed. Depending on the status of the disease, para-aortic lymph node dissection may or may not be performed

Modified radical hysterectomy is the routine procedure for stage IA2 invasive cervical cancer. It is positioned between total and radical hysterectomy. Bilateral oophorectomy is usually performed. Pelvic or para-aortic lymph node dissection may or may not be performed depending on the status of the disease

Radical hysterectomy is the routine procedure for stage IB2 invasive cervical cancer. It involves the removal of the uterus, cervix, and the upper one-third of the vagina along with the cardinal ligament. Additionally, pelvic lymph node dissection and biopsy of para-aortic lymph nodes must be performed. Bilateral oophorectomy is usually performed

^a^Data missing: *n* (%) → 3 (1%)

^b^Data missing: *n* (%) → 34 (11.2%)

^c^Data missing: *n* (%) → 2 (1%)

*BMI* body mass index, *FIGO* International Federation of Gynecology and Obstetrics, *LN* lymph node, *CI* confidence interval

 The relationships between risk factors were modeled in the second component. Since all endogenous risk factors were binary, as shown in Fig. 3, logistic regression models were used to evaluate the interrelationship among risk factors. Standard treatment for each gynecologic malignant tumor includes surgery, chemotherapy, radiotherapy, or any combination, as shown in Table 2. Thus, although standard therapy differs depending on the type of cancer, the results in Table 3 indicate that cancer type has no influence on LLL. The odds of receiving radiotherapy were 42.4 times higher in patients with cervical cancer than in patients with other types of cancer (95% CI 29.5–60.9). Similarly, the odds of receiving radiotherapy were 6.4 times higher among patients with FIGO stages III–IV than in those with stages I–II (95% CI 4.4–9.3). Table 3 shows the incidence of LLL at LN excision sites was 17.5% (29/166) in patients with inguinal LN dissection (95% CI 0.87–3.04), 17 % (34/200) in patients with pelvic LN dissection (95% CI 0.86–3.53), and 18% (15/83) in patients with para-aortic LN dissection (95% CI 0.75–2.60). However, as shown in Table 4, the probability of removing each LN with 28 or more LN resections compared to less than 28 is 5.9 times higher with inguinal LN (95% CI 4.4–8.0). It is 4.0 times higher in pelvic LN (95% CI 2.7–5.9) and 2.8 times higher in para-aortic LN (95% CI 2.1–3.6). Similarly, patients with cervical cancer were 1.6 times more likely than patients with non-cervical cancer to have 28 or more LN resections (95% CI 1.3–2.1). There was no difference in the incidence of LLL by individual site, but it was higher in cases of 28 or more. Total LN dissections indicates the intensity (thoroughness) of the dissection, and it was confirmed that inguinal LN dissection was performed in cases where thorough dissection was required. A high proportion of LLL was observed when inguinal LN dissection was performed indirectly. Other odds ratios are shown in Table 4. All parameter estimates in Table 4 were obtained simultaneously based on GSEM.

**Table 4:** Estimate of model parameter on the risk factors model

Component 1 outcome (time to event)	Coefficient	Hazard ratio	95% CI	*p* value
Direct effect of "lymphedema onset"				
FIGO (III–IV)	0.824	2.3	1.2–4.2	0.009
Radiotherapy (performed)	0.804	2.2	1.1–4.5	0.027
Number of LN dissection (≥ 28)	0.64	1.9	1.0–3.5	0.037

Component 2 outcome (yes/no)	Coefficient	Odds ratio	95% CI	*p* value
Direct effect of "FIGO stage (III–IV)"				
LN metastasis (positive)	2.722	15.2	11.4–20.2	< 0.001
Direct effect of "Radiotherapy (performed)"				
Type of cancer (cervical cancer)	3.748	42.4	29.5–60.9	< 0.001
FIGO (III–IV)	1.854	6.4	4.4–9.3	< 0.001
Direct effect of "Number of LN resection (≥ 28)"				
Inguinal LN dissection (+)	1.783	5.9	4.4–8.0	< 0.001
Pelvic LN dissection (+)	1.396	4.0	2.7–5.9	< 0.001
Para-aortic LN dissection (+)	1.012	2.8	2.1–3.6	< 0.001
Type of cancer (cervical cancer)	0.467	1.6	1.3–2.1	< 0.001

*LN* lymph node, *FIGO* International Federation of Gynecology and Obstetrics, *CI* confidence interval

## Discussion

The accumulation of scientific information on the developmental history of LLL has been delayed because priority was given to treating gynecological cancer. Hence, this study mainly focused on reporting the incidence of LLL development and its clinical risk factors based on sound scientific methods. This study differs from previous reports in that the risk factors for LLL development in patients treated for gynecologic cancer (cervical cancer, endometrial cancer, and ovarian cancer including fallopian tube cancer) were examined without limiting the treatment method. Although there are differences in standard treatment depending on the type of cancer, the results of the univariate analysis did not directly affect the occurrence of LLL according to type of cancer. Therefore, we analyzed the risk factors directly related to LLL occurrence, regardless of the type of cancer. As a result, the number of LNs dissected (≥ 28) was a significant risk factor for LLL development regardless of site and extent of LN dissection. Nine out of 54 cases of LLL showed recurrence. However, the relationship between recurrence as a pelvic mass and occurrence of LLL could not be denied in only one case of recurrence. Therefore, globally, it can be presumed that the occurrence of LLL is due to treatment. This result supports the notion that sentinel LN biopsies result in lower incidence of LLL by not performing unnecessary LN dissections. Sentinel LN is defined as a LN where cancer cells reach LN metastasis through the tumor and the lymph vessel connected to it. Therefore, if cancer metastasis has not occurred in the sentinel LN, it is believed that there is a high possibility that it has not spread to other LNs, and there is no other metastasis. Therefore, to reduce the number of LN resections recognized as a risk factor for the development of LLL less than 28, the presence of metastasis to sentinel LNs [24, 25] is confirmed, and the significance of dissection biopsy is high. In the future, sentinel LN biopsy should be actively introduced to reduce the incidence of LLL. According to the literature, there are limited records of patients after surgery. Although there are reports on the incidence and timing of postoperative LLL, there is no clear report on the incidence and timing of LLL in gynecologic cancer treatment after initial treatment, including treatments other than surgery.

For example, Kim et al. [5] reported 1-year and 3-year prevalence rates of 42.7% and 78.7%, respectively, with a median onset time of 11 months. Our 5-year follow-up records indicated similar prevalence rates based on the Kaplan–Meier estimate. Additionally, Graf et al. [26] reported an estimated prevalence of LLL of 32% 1 year after surgery and 58% 8 years after surgery. Hareyama et al. [27] reported that the cumulative incidence of patients who underwent lymphadenectomy was 12.9% at 1 year, 20.3% at 5 years, and 25.4% at 10 years. The reported risk factors from many previous studies [3–21, 26, 27] were mainly based on postoperative patients with limited and specific treatments. In contrast to the findings from previous studies, we evaluate risk factors based on all patients who received initial treatment for gynecologic cancer, where initial treatment was defined as either surgery, chemotherapy, radiotherapy, or any combination therapy. From the results of this study, regardless of the type of treatment, it is clear that LLL occurs in about 15% of patients within 2 years of initial treatment. Therefore, accurate information can be provided to patients.

Previous studies [3, 6, 9–21] have reported postoperative radiotherapy and the number of LN dissections as risk factors for LLL. However, some studies have reported that the location of the LN is a risk factor. For example, Kuroda et al. [4] studied pelvic lymphadenectomy (PLA) in patients with or without para-aortic lymphadenectomy (PALA) and reported BMI (≥ 25), PLA + PALA, and postoperative radiation therapy as the risk factors. In contrast, Ohba et al. [6] reported that suprafemoral node dissection and postoperative radiation therapy are risk factors for the development of LLL. However, a study on postoperative cervical cancer patients reported that pelvic and para-aortic lymphadenectomy and the number of LNs are not risk factors. Todo et al. [8] examined the effect of removing circumflex iliac nodes up to the distal external iliac nodes (CINDEIN) in patients with uterine corpus malignancies. It was reported that adjuvant radiation therapy, resection of more than 31 LN, and removal of CINDEIN are risk factors for the development of LLL. These findings were mainly based on postoperative patients who received limited and specific treatment. Unlike previous studies, this study included all patients who received initial treatment for gynecologic cancer, regardless of surgery. This includes patients who had been treated with chemotherapy or radiation therapy and who had not undergone surgery. Therefore, it is speculated that lymphadenectomy did not directly affect the development of LLL.

As shown in Table 2, although the maximum number of LN dissections in cervical cancer is small, compared to those in endometrial cancer and ovarian cancer, the average number of LN dissections in cervical cancer was higher than that in cervical cancer and ovarian cancer. The reason is that pelvic LN dissection is usually performed in cervical cancer, but in endometrial/ovarian cancer, pelvic LN dissection or para-aortic LN dissection may not be performed for low-risk patients who do not have cancer metastasis at each LN site. In some cases, LN dissection was omitted, and only LN biopsy was performed, and the reason for this study may be that there were many low-risk patients with endometrial/ovarian cancer. LN biopsy randomly collects one or two LNs to see if there is cancer LN metastasis. However, LN dissection is performed when the cancer is likely to have spread to it as the LNs in the cancerous area are removed for the purpose of preventing recurrence; the number of LN dissection inevitably increases. As shown in Table 3, LN excision and cancer type at each site did not directly affect the onset of LLL. However, as shown in Table 4, when each LN excision site and cancer type had 28 or more LN excisions it had an indirect effect on LLL. Although detailed data are not shown here, differences in surgery by cancer type were not found to directly affect the development of LLL (Fisher’s exact test *p* = 0.57). However, as shown in Table 4, the incidence of LLL was high when there were 28 or more LN dissections. Therefore, I thought that the model in Fig. 3 was appropriate.

The novelty of our findings on risk factors may be explained by the different analytical approaches. Kimura et al. [1] suggested the importance of considering the relationship between risk factors from a clinical viewpoint. As risk factors were often highly correlated, multivariate Cox regression analyses were likely faced with the issue of multi-collinearity. The generalized structural equation model proved to be a useful analytical tool to help avoid this difficulty and examine the relationship among risk factors simultaneously.

The limitations of our study were single-center medical record data and the number of patients may have been insufficient to confirm the effects of cancer types. Another limitation is the lack of data on the severity and extent of LLL development.

In this study, the risk factors directly affecting LLL development were FIGO progression stage (III–IV), number of LNs dissected (≥ 28), and radiotherapy (performed). Simultaneous examination of the relationship between LLL and risk factors, and among the risk factors using GSEM, enabled us to construct a clinical model of LLL development, which can be used in the clinical setting. It will also provide information to alleviate the incidence of LLL and improve patient QOL.

## Appendix: Fitting the cox proportional hazard model via the generalized structural equation model

Let *T*_*i*_ be onset time of lymphedema for *i*th patient $\left( {i = 1, \ldots ,n} \right)$. Onset time is interval censored if we only know event occured within time interval $\tau_{r - i} < T_{i} \le \tau_{r} \, (r = 1, \ldots ,m)$. If event or censoring occured in time interval *a*, hazard function *h*(*a*) for i-th subject is defined as $h(a_{i} ) = P(T_{i} = a_{i} \left| {T_{i} > a_{i} - 1} \right.)$.

Then, *i*th subject's contribution to the likelihood, *L*_*i*_, is

$$ L_{i} = \prod\limits_{r = 1}^{{a_{i} }} {h(r)^{{Y_{ir} }} (1 - h(r))^{{1 - Y_{ir} }} } $$

hear, *Y*_*ir*_ is an indicator variable defined as *Y*_*ir*_ = 0 if r < *a*_*i*_ and $Y_{ir} = \delta_{{\text{i}}}$ if *r* = *a*_*i*_ where $\delta_{i}$ is censoring indicator (= 1 if onset lymphedema, otherwise = 0).

It can be shown that $\log \left\{ { - \log h(r|X_{i} )} \right\} = \alpha_{r} + X_{i}^{\prime } \beta$ if we assume proportional hazard model $h(r|X_{i} ) = h_{0} (r)\exp (X_{i}^{\prime } \beta )$ where *X*_*i*_ is a vector of risk factors, $\alpha_{r} = \log \left\{ {\log S_{0} (r) - \log S_{0} (r - 1)} \right\}$ and *S*_0_(*r*) = baseline survival function [28, 29].

Let $\mu_{ir}$ is expectation of *Y*_*ir*_, $E(Y_{ir} ) = \mu_{ir}^{(Y)}$, then survival analysis of lymphedema is modeled as $g_{1} {(}\mu_{ir}^{(Y)} ) = \alpha_{r} + X_{i}^{\prime } \beta$ where *g*_1_ is complimentary log–log link function.

Effect of exogenous risk factor *W*_*i*_ on endogenous risk factors *X*_*i*_ will be modeled as $g_{2} \left( {\mu_{i}^{(X)} } \right) = \gamma^{\prime}W_{i}$ where $\mu_{i}^{(X)}$ is mean vector of *X*_*i*_ and g_2_ is an appropriate link function for *X*. $\left\{ {g_{1} \left( {\mu_{ir}^{(Y)} } \right),g_{2} \left( {\mu_{i}^{(X)} } \right)} \right\}$ are simultaneously modeled under generalized structural equation models [30].

## References

[1] Kimura E (2013). A literature review about risk factor of lymphoedema. Lymph RAP.

[2] Biglia N, Zanfagnin V, Daniele A (2017). Lower body lymphedema in patients with gynecologic cancer. Anticancer Res.

[3] Akita S, Mitsukawa N, Rikihisa N (2013). Early diagnosis and risk factors for lymphedema following lymph node dissection for gynecologic cancer. Plast Reconstr Surg.

[4] Kuroda K, Yamamoto Y, Yanagisawa M (2017). Risk factors and a prediction model for lower limb lymphedema following lymphadenectomy in gynecologic cancer: a hospital-based retrospective cohort study. BMC Womens Health.

[5] Kim JH, Choi JH, Ki EY (2012). Incidence and risk factors of lower-extremity lymphedema after radical surgery with or without adjuvant radiotherapy in patients with FIGO stage I to stage IIA cervical cancer. Int J Gynecol Cancer.

[6] Ohba Y, Todo Y, Kobayashi N (2011). Risk factors for lower-limb lymphedema after surgery for cervical cancer. Int J Clin Oncol.

[7] Beesley V, Janda M, Eakin E (2007). Lymphedema after gynecological cancer treatment: prevalence, correlates, and supportive care needs. Cancer.

[8] Todo Y, Yamazaki H, Takeshita S (2015). Close relationship between removal of circumflex iliac nodes to distal external iliac nodes and postoperative lower-extremity lymphedema in uterine corpus malignant tumors. Gynecol Oncol.

[9] Beesley VL, Rowlands IJ, Hayes SC (2015). Incidence, risk factors and estimates of a woman's risk of developing secondary lower limb lymphedema and lymphedema-specific supportive care needs in women treated for endometrial cancer. Gynecol Oncol.

[10] Yost KJ, Cheville AL, Al-Hilli MM (2014). Lymphedema after surgery for endometrial cancer: prevalence, risk factors, and quality of life. Obstet Gynecol.

[11] Todo Y, Yamamoto R, Minobe S (2010). Risk factors for postoperative lower-extremity lymphedema in endometrial cancer survivors who had treatment including lymphadenectomy. Gynecol Oncol.

[12] Tada H, Teramukai S, Fukushima M (2009). Sasaki H: Risk factors for lower limb lymphedema after lymph node dissection in patients with ovarian and uterine carcinoma. BMC Cancer.

[13] Hayes SC, Janda M, Ward LC (2017). Lymphedema following gynecological cancer: results from a prospective, longitudinal cohort study on prevalence, incidence and risk factors. Gynecol Oncol.

[14] Halaska MJ, Novackova M, Mala I (2010). A prospective study of postoperative lymphedema after surgery for cervical cancer. Int J Gynecol Cancer.

[15] Fuller J, Guderian D, Kohler C (2008). Lymph edema of the lower extremities after lymphadenectomy and radiotherapy for cervical cancer. Strahlenther Onkol.

[16] Mendivil AA, Rettenmaier MA, Abaid LN (2016). Lower-extremity lymphedema following management for endometrial and cervical cancer. Surg Oncol.

[17] Abu-Rustum NR, Alektiar K, Iasonos A (2006). The incidence of symptomatic lower-extremity lymphedema following treatment of uterine corpus malignancies: a 12-year experience at Memorial Sloan-Kettering Cancer Center. Gynecol Oncol.

[18] Ki EY, Park JS, Lee KH (2016). Incidence and risk factors of lower extremity lymphedema after gynecologic surgery in ovarian cancer. Int J Gynecol Cancer.

[19] Mitra D, Catalano PJ, Cimbak N (2016). The risk of lymphedema after postoperative radiation therapy in endometrial cancer. J Gynecol Oncol.

[20] Hopp EE, Osborne JL, Schneider DK (2016). A prospective pilot study on the incidence of post-operative lymphedema in women with endometrial cancer. Gynecol Oncol Rep.

[21] Achouri A, Huchon C, Bats AS (2013). Complications of lymphadenectomy for gynecologic cancer. Eur J Surg Oncol.

[22] Kunitake T, Kakuma T, Ushijima K (2018). Lymphedema development in the lower extremities of patients with gynecological malignancies. J Lymphedema Res.

[23] Lin JH, Lee WC (2016). Complementary log regression for sufficient-cause modeling of epidemiologic data. Sci Rep.

[24] Bodurtha Smith AJ, Fader AN, Tanner EJ (2017). Sentinel lymph node assessment in endometrial cancer: a systematic review and meta-analysis. Am J Obstet Gynecol.

[25] Wang T, Hu Y, He Y (2019). A retrospective validation study of sentinel lymph node mapping for high-risk endometrial cancer. Arch Gynecol Obstet.

[26] Graf N, Rufibach K, Schmidt AM (2013). Frequency and risk factors of lower limb lymphedema following lymphadenectomy in patients with gynecological malignancies. Eur J Gynaecol Oncol.

[27] Hareyama H, Hada K, Goto K (2015). Prevalence, classification, and risk factors for postoperative lower extremity lymphedema in women with gynecologic malignancies: a retrospective study. Int Gynecol Cancer.

[28] Rabe-Hesketh S, Skrondal A (2005). Multilevel and longitudinal modeling using stata.

[29] Thompson WA (1977). On the treatment of grouped observations in life studies. Biometrics.

[30] STATA Structural Equation Modeling Reference manual release 13 https://www.stata.com/manuals13/sem.pdf. Accessed 1 Sept 2018

